# Multifocal osteonecrosis secondary to occupational exposure to aluminum

**DOI:** 10.1590/1413-785220172503170760

**Published:** 2017

**Authors:** Jorge Henrique Assunção, Eduardo Angeli Malavolta, Mauro Emilio Conforto Gracitelli, Renée Zon Filippi, Arnaldo Amado Ferreira

**Affiliations:** 1 Universidade de São Paulo, Faculdade de Medicina, Hospital das Clínicas, Instituto de Ortopedia e Traumatologia, Grupo de Ombro e Cotovelo, São Paulo, SP, Brazil

**Keywords:** Osteonecrosis, Occupational injuries, Shoulder, Hip, Knee

## Abstract

Multifocal osteonecrosis is a rare disease; chronic use of corticosteroids is considered the main risk factor. Patients with chronic renal failure can develop aluminum toxicity, which can lead to osteomalacia and encephalopathy. An association between osteonecrosis and aluminum toxicity has been reported among patients with dialytic renal insufficiency. Occupational exposure to aluminum rarely causes lung disease and no cases of bone lesions resulting from exposure to this metal have been reported. In this manuscript, we describe a novel case of a patient with multifocal osteonecrosis associated with chronic occupational exposure to aluminum. ***Level of Evidence IV, Case Report.***

## RESUMO

A osteonecrose multifocal é uma doença rara. O uso crônico de corticoides é considerado seu principal fator de risco. Em pacientes com insuficiência renal crônica é descrita a intoxicação por alumínio, causando osteomalácia e encefalopatia. A relação entre osteonecrose e toxicidade pelo alumínio já foi relatada em estudos que envolveram pacientes com insuficiência renal dialítica. A exposição ocupacional ao alumínio pode, raramente, ocasionar doenças pulmonares. Não existem casos relatados de lesões ósseas decorrentes da exposição a esse metal. Descrevemos neste artigo, o relato inédito de um paciente com osteonecrose multifocal associada à exposição ocupacional crônica ao alumínio. ***Nível de Evidência IV, Relato de Caso.***

## INTRODUCTION

Osteonecrosis is a common orthopedic disease^1^ and tends to affect the hip.^2^ Multifocal involvement has a prevalence of only 3%.^3^ Prominent among occupational causes is dysbaric disease.^4^ Occupational exposure to aluminum is a rare cause of disease,^5^ and its relationship with pneumoconiosis has been established.^5^ ^,^ ^6^ In this paper we report an unprecedented case of multifocal osteonecrosis secondary to chronic occupational exposure to aluminum.

## METHODS

Black male patient, 39 years old, was evaluated for the first time in our service in 2008. He presented polyarthralgia involving knees, hips and shoulders, which started four years prior. At the time of the consultation, the pain was debilitating. He worked for eight years in a plant refining bauxite and producing aluminum. His job was to open packages containing solid material and empty them into a tank, where a chemical reaction occurred. During his work, he used glasses, a filter mask, ear plugs, boots and a uniform with long cotton sleeves and gloves. He states he did not use a helmet. He did not come into contact with ionizing radiation or a hyperbaric chamber.

**Figure 1:**
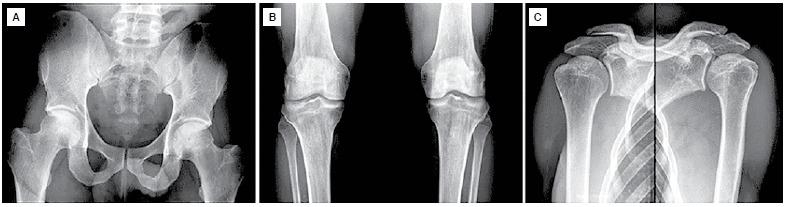
X-rays of hips (A), knees (B) and shoulders (C) showing preserved joint congruity.

**Figure 2:**
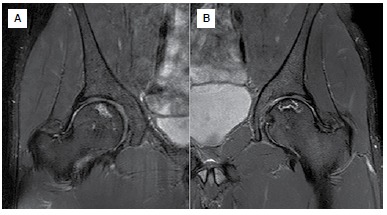
MRI of the hips showing bilateral involvement without joint collapse: (A) right, (B) left.

X-rays of the hips, knees and shoulders did not show alterations. (Figure 1) MRI scans showed signs of osteonecrosis. (Figures 2-^4^) In the hips and shoulders, the location was subchondral, while in the knees it was predominantly located in the metaphysis of the femur as well as the tibia. Collapse was not observed in any of the joints. According to the visual analogue pain scale (VAS), the patient scored nine points in the right hip, knee and shoulder and seven in the left hip, knee and shoulder. Range of motion was complete in all joints except the patient's right shoulder, which had 150° elevation, internal rotation to L5 and external rotation of 40°.

**Figure 3:**
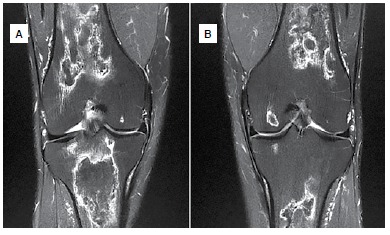
MRI of the knees showing bilateral involvement without joint collapse: (A) right, (B) left.

**Figure 4:**
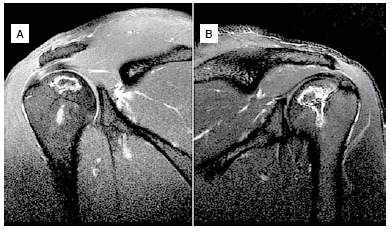
MRI of the shoulders showing bilateral involvement without joint collapse: (A) right, (B) left.

The patient was subjected to extensive laboratory testing. No changes were seen in kidney, liver, thyroid, pancreatic, or parathyroid function. Electrolytes (sodium, potassium, calcium, phosphorus and magnesium) were at normal concentrations, as well as vitamin D, cholesterol, triglycerides and plasma proteins. The blood count did not show any abnormalities. Inflammatory and rheumatological tests were negative.

Predisposing factors for thromboembolic phenomena (coagulation, platelet concentration, antiphospholipid syndrome, factor V Leiden, anti-cardiolipin) were also normal. Serology for hepatitis, HIV and HTLV was negative.

Bone marrow biopsy showed no alterations. Electrophoresis of hemoglobin showed 62.1% hemoglobin A1, 2.3% hemoglobin A2 and 35.6% hemoglobin S (sickle cell trait). The bone lesion present in the metaphyseal region of the left femur was biopsied, showing bone infarction.

**Figure 5:**
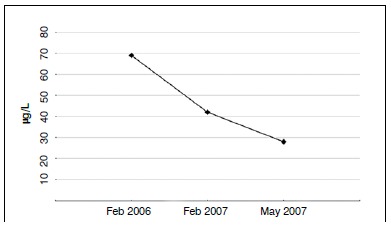
Graph showing patient's plasma aluminum concentration.

**Figure 6:**
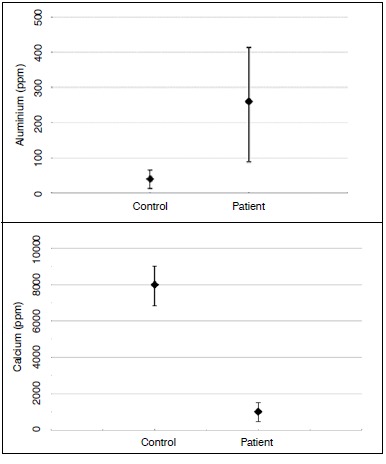
Graph showing high concentration of aluminum and low concentration of calcium in the bone tissue compared to a healthy sample control.

The patient had plasma aluminum above normal levels in all samples. (Figure 5) A new biopsy was performed in the iliac crest, which showed a high concentration of aluminum and a low calcium concentration in relation to a healthy sample control. X-ray fluorescence spectrometry was employed. (Figure 6)

**Figure 7:**
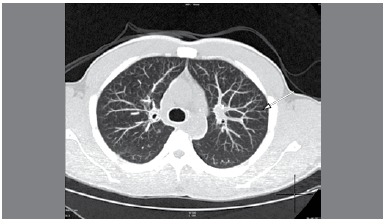
CT scan of the chest showing centrilobular micronodules (arrows).

The patient did not report respiratory symptoms. Tomography of the chest revealed the presence of centrilobular micronodules, (Figure 7) both calcified and uncalcified. A lung biopsy collected via bronchoscopy showed normal tissue and bronchoalveolar lavage did not show the presence of fungi or mycobacteria.

Conservative treatment was indicated, with pain relief and physiotherapy. The patient currently exhibits level five pain in the lower limbs and four in the upper limbs and full range of motion in the involved joints. The patient uses analgesics regularly and chose not to undergo decompression in the foci of osteonecrosis. X-rays still show no signs of collapse after six years of follow-up. The patient has remained on disability leave from his work since 2008. The study was approved by the institutional review board under process number 1113.

## DISCUSSION

A variety of causes have been described for osteonecrosis. Among extrinsic or iatrogenic causes are decompression sickness,^1^ ^,^ ^4^ alcohol consumption^1^ ^,^ ^2^and chronic use of corticosteroids.^1^ ^,^ ^2^

Multifocal involvement, defined as occurring in three or more places, is rare. In a study involving 1056 patients with osteonecrosis, LaPorte et al.^3^ found only 3% with multifocal involvement. The average number of affected sites was 6.3 and 77% of the joints did not collapse. Another multicenter study^7^ involving 101 patients with osteonecrosis found progressive involvement of the femoral head in 100% of cases, the knee in 96%, the shoulder in 80% and the ankle in 44%. Bilaterality was commonly found in the hip (98%), knees (86%) and shoulder (83%). Most of the injuries (69%) were diagnosed in pre-collapse stage. The case described here presented involvement in six joints (hips, knees and shoulders), all without collapse.

Previous use of corticosteroids represents 91% of the causes of multifocal osteonecrosis.^7^ Other less common causes are alcoholism,^1^ chemotherapy,^8^ sickle cell disease,^9^ rheumatological diseases,^3^ coagulation disorders,^10^ inflammatory bowel disease,^11^ and HIV infection.^12^ In the case described here, the patient was negative for all these risk factors.

The presence of the sickle cell trait, as seen in our patient, has been reported in association with osteonecrosis of the hip.^13^ However, the evidence is insufficient to determine a significant association.^14^ Dorwart et al.,^15^ in a larger study on this subject, observed that the occurrence of osteonecrosis was not higher in the 114 patients evaluated in comparison with controls. We believe that the sickle cell trait was not a determining factor in the development of the multiple osteonecrotic foci.

In patients with chronic renal failure, aluminum poisoning resulting from hemodialysis fluids and/or oral prophylactic use of phosphate chelating agents has been described as causing osteomalacia and encephalopathy.^16^ The relationship between osteonecrosis and aluminum toxicity has been reported in only two studies involving patients with dialytic renal failure.^17^ ^,^ ^18^ However, we found no reports associating the occurrence of osteonecrosis with occupational exposure to aluminum. In 2007, Krewsky et al.^5^ published a systematic review of the risks aluminum poses to health. These authors did not refer to any bone complications resulting from occupational exposure in their study. Willhite et al.^6^ updated this systematic review, also without reporting osteonecrosis as a complication.

The relationship between aluminum exposure and pneumoconiosis is well established, however.^5^ ^,^ ^6^ ^,^ ^19^ Kraus et al.^19^ reported data on 62 workers involved with the production of aluminum powder with median exposure of 123 months. These authors found nodular centrolobular opacity in tomography in 24.2% of their sample and 6.5% reported effort dyspnea. They also observed that plasma and urine concentrations of aluminum are correlated to labor risks. Our case presented calcified and non-calcified centrilobular micronodules in tomography. Despite these findings, the patient denied present or past respiratory discomfort. We believe that the high concentration of aluminum in the bloodstream, after inhalation or skin absorption, could be the cause of osteonecrosis in the case in question. Aluminum inhibits osteoid tissue calcification of the trabecular bone,^17^ ^,^ ^18^ and consequently the resulting osteomalacia makes the bone tissue more fragile and susceptible to osteonecrosis from microtrauma.^18^ The patient was exposed to aluminum until 2005, when he took disability leave from his work. Three plasma aluminum levels were taken, one in 2006 and two in 2007. In all tests the concentrations were high and gradually decreased. Another factor that contributes to our hypothesis was the high aluminum level seen in the patient's bone tissue via x-ray fluorescent spectrometry.

Pre-collapse osteonecrosis can be treated conservatively or with surgery in order to relieve pain and prevent collapse.^20^ Decompression of the focus of the osteonecrosis is an effective procedure for treating early stages of osteonecrosis of the hip, knee and shoulder,^3^ although there is no consensus on the indications for this procedure.^20^ When joint collapse or secondary arthrosis has already occurred, joint arthroplasty is the recommended treatment.^3^ The case reported herein was treated conservatively, with partial improvement of pain and no collapse in 6 years of follow-up. Nevertheless, the patient uses opioids regularly. Surgical decompression was indicated for the foci of the osteonecrosis, but the patient opted for non-surgical treatment despite the severity of painful symptoms.

## CONCLUSION

In this article we describe an unpublished report of a patient with multifocal osteonecrosis associated with chronic occupational exposure to aluminum.
